# Dynamic Changes in the Follicular Transcriptome and Promoter DNA Methylation Pattern of Steroidogenic Genes in Chicken Follicles throughout the Ovulation Cycle

**DOI:** 10.1371/journal.pone.0146028

**Published:** 2015-12-30

**Authors:** Guiyu Zhu, Yong Mao, Wendi Zhou, Yunliang Jiang

**Affiliations:** 1 Department of Biology Science and Technology, Taishan University, Taian 271021, China; 2 Shandong Provincial Key Laboratory of Animal Biotechnology and Disease Control and Prevention, College of Animal Science and Veterinary Medicine, Shandong Agricultural University, Taian 271018, China; 3 Department of Gynecology, Taian Materal and Child Health Hospital, Taian 271021, China; John Hopkins University School of Medicine, UNITED STATES

## Abstract

The molecular mechanisms associated with follicle maturation and ovulation are not well defined in avian species. In this study, we used RNA-seq to study the gene expression profiles of the chicken follicles from different developmental stages (pre-hierarchical, pre-ovulatory and post-ovulatory). Transcriptomic analysis revealed a total of 1,277 and 2,310 genes were differentially expressed when follicles progressed through the pre-hierarchical to hierarchical and pre-ovulatory to post-ovulatory transitions, respectively. The differentially expressed genes (DEG) were involved in signaling pathways such as adherens junction, apoptosis and steroid biosynthesis. We further investigated the transcriptional regulation of follicular steroidogenesis by examining the follicle-specific methylation profiles of Star (steroidogenic acute regulatory protein), Cyp11a1 (cytochrome P450, family 11, subfamily a, polypeptide 1) and Hsd3b (hydroxy-delta-5-steroid dehydrogenase, 3 beta- and steroid delta-isomerase 1), genes encoding the key enzymes for progesterone synthesis. The varied patterns of DNA methylation in proximal promoters of Star and Cyp11a1but not Hsd3b in different follicles could play a major role in controlling gene expression as well as follicular steroidogenic activity. Finally, the promoter-reporter analysis suggests that TGF-β could be involved in the regulation of Hsd3b expression during ovulation. Together, current data not only provide novel insights into the molecular mechanisms of follicular physiology in chicken follicles, but also present the first evidence of epigenetic regulation of ovarian steroidogenesis in avian species.

## Introduction

The domestic fowl provides a unique model for studying molecular and cellular mechanisms during follicular development, ovulation and regression. Unlike mammalian counterparts, the single left ovary of the hen contains follicles of various sizes and developmental stages. Generally, a functional mature Leghorn hen ovary contains thousands of quiescent primordial follicles, hundreds of growing pre-hierarchical follicles (small white follicles and small yellow follicles), 5–6 large yellow pre-ovulatory follicles that are 9–40mm in diameter and 2–4 post-ovulatory follicles devoid of oocyte[1]. Therefore, the resting primordial follicles, pre-hierarchical growing follicles, pre-ovulatory follicles and post-ovulatory follicles are represented simultaneously in one reproductively active ovary.

All hen follicles, irrespective of size or developmental stage, are exposed to similar levels of gonadotropins and steroids present in the ovary. Nevertheless, only one single specific follicle is selected per day from the cohort of pre-hierarchical follicles into the pre-ovulatory queue to begin rapid growth until ovulation. Therefore, the whole reproductive cycle requires finely controlled endocrine, paracrine and autocrine factors to regulate the streamlined hierarchical follicles of all different stages within the same ovary. Furthermore, unlike corpus luteum formation in mammals, the post-ovulatory follicles disappear within days, as it is difficult to find the 4^th^ or 5^th^ post-ovulatory follicles [2,3]. This rapid degradation is necessary for the next ovulation as well as the new hierarchical recruitment[4]. However, it seems that the post-ovulatory follicles were also required for successful oviposition and nesting behaviors [5].

Once the follicle is selected for ovulation, it rarely goes to alternative fates. Therefore, researchers focused mainly on the signaling pathways related to the selection of pre-hierarchical follicles into the pre-ovulatory follicle hierarchy[6]. Less attention has been devoted to elucidate the molecular mechanisms regulating other processes of the complete course, such as the development of the follicles to attain maturity, the control of the ovulation process as well as the degradation of post-ovulatory follicles. The morphological and physiological reorganization of the developing and regressing chicken follicles is preceded by a profound and well-orchestrated modulation of gene expression. Comparative gene expression profiling in different follicles can provide information for understanding the molecular mechanisms that control the follicle selection, development, ovulation and regression.

Given that the follicles undergo dramatic alterations in phenotype throughout the ovulatory cycle, of particular interest are the cell signaling and associated transcriptional mechanisms that regulate the transitions of pre-hierarchical to hierarchical and pre-ovulatory to post-ovulatory follicles. In the current study, gene expression profiles of pre-hierarchical follicles (small white follicles), pre-ovulatory follicles and post-ovulatory follicles were obtained by RNA-seq and differentially expressed genes (DEG) were identified by further pairwise comparisons. Gene pathway analysis indicates that these DEGs are involved in different signaling pathways such as adherens junction, apoptosis and steroids biosynthesis. Among the ovarian steroid hormones, the progesterone is the basic steroid that many other steroid hormones derive from and plays an essential role in avian reproduction[7]. Therefore, we further examined the transcriptional regulation of genes encoding for the key enzymes in progesterone synthesis in chicken follicles and evidence suggests that the combined genetic and epigenetic alterations in steroidogenic genes could play a major role in controlling chicken progesterone synthesis.

## Materials and Methods

### Animal sampling, follicle collection and cell culture

Sexually mature (older than 23 weeks) Hy-Line Brown hens were collected from a local research farm affiliated with Shandong Agricultural University. All animals had free access to water and feed. The hens were housed in separate cages under a daily light period of 14 h and the laying events were recorded by checking the cage every 1–2 h during the light period to determine the regular laying sequence. To obtain follicles from the ovaries, hens in the middle of a laying sequence were sacrificed by decapitation immediately after oviposition. Follicles at different developmental stages including pre-hierarchal small white follicles (SWF, 2–4 mm), the largest pre-ovulatory follicles (F1) and post-ovulatory follicles (POF1) were collected and stored in liquid nitrogen for RNA isolation. The yolk in the SWF and F1 follicles was carefully removed with a syringe and a 25-gauge needle before snap frozen. The granulosa cells were isolated as described previously [8,9]. In brief, the second largest (F2), third largest (F3), and fourth largest (F4) follicles were isolated and punctuated with sterile needles to remove the yolk. The isolated granulosa sheets were washed in pre-warmed Hanks' balanced salt solution and then dispersed by treatment with 0.3% collagenase at 37°C for 10 min with gentle agitation in a flask. After centrifugation, the cells were suspended in culture medium (M199 [Gibco] with 2.5% fetal bovine serum and 1% penicillin/streptomycin) and subsequently seeded in 24-well culture plates at a density of 2 ×10^5^/well. The number of viable cells (>90%) was estimated using Trypan blue. Cells were cultured at 39°C in a water-saturated atmosphere of 5% CO_2_ for 24 h. Then the serum-free medium was used for the subsequent experiments. All animal experiments were approved by the Institutional Animal Care and Use Ethics Committee of Shandong Agricultural University (Permit Number: 2001002) and performed in accordance with the “Guidelines for Experimental Animals” of the Ministry of Science and Technology of China.

### RNA isolation and RNA-Seq library preparation

Total RNA from follicle samples including SWF, F1 and POF1 follicles was extracted using Trizol reagent (Invitrogen) according to manufacturer’s instruction. The RNA samples from 6 different hens in the same group were pooled together based on an equal RNA quantity. Poly(A) RNA purification, cDNA synthesis, tag preparation and RNA-Seq were performed by technicians in line with specified experimental process at Beijing Genome Institute (BGI) (Shenzhen, China). Briefly, mRNA are enriched through beads of Oligo (dT) and transferred into double-stranded cDNA *via* reverse transcription. cDNA is digested with NlaIII which cut off CATG sites and Illumina adaptor 1 is ligated to the 5' end of fragments. Then, Mmel is used to digest at 17 bp downstream of CATG site and Illumina adaptor 2 is ligated at 3' end. Finally, 95 bp fragments produced by PCR amplification are purified through 6% TBE PAGE and sequenced using Illumina HiSeq 2000 system. Prior to analysis, the primary sequenced data were filtered to remove raw reading frames containing adapter sequences, reads containing poly-N sequences and low-quality reading frames from raw data. The clean reading frames were mapped to chicken genome assembly Gallus_gallus-4.0 (http://www.ncbi.nlm.nih.gov/assembly/317958). Differentially expressed genes were identified according to [10]. DEGs were defined according to the following criteria: (1) p ≤ 0.05, (2) average read count of ≥10 in at least one experimental group, and subsequently used false discovery rate (FDR) ≤0.001, (3) fold-change threshold ≥2. The biological significance of the DEGs was assessed by GO classification (http://wego.genomics.org.cn) and KEGG pathway analysis [11,12].

### Real-time Quantitative PCR validation

Total RNA from the different follicles was extracted using Trizol and total RNA from the cultured granulosa cells was isolated with an RNeasy Mini Kit (Qiagen). The RNA samples were assessed by gel electrophoresis, and the 260/280 optical density ratios of 1.8 or higher were obtained. The cDNA was synthesized using AccuScrip High Fidelity Reverse Transcriptase (Stratagene) according to the manufacturer's instructions. The total reaction volume of 20 μl contained 3 μg total RNA, 2 μl RT-buffer, 1 μl oligo-d(T) primer (10 μM), 1 μl dNTPs (25 mM each), 2 μl dithiothreitol (100 mM), 1 μl reverse transcriptase, and RNase-free-water. Real-time quantitative PCR (qPCR) was performed using SYBR premix Ex Taq (TaKaRa). The total reaction volume was 15 μl, including 2 μl of cDNA template, 7.5 μl of Master Mix, 0.3 μl SYBR Green Rox, 1 μl each of the forward and reverse primers (10 μM), and water. The program was 95°C for 30 sec followed by 40 cycles of 95°C for 5 sec, 56°C for 30 sec, and 72°C for 30 sec. Melting curves were used to confirm the specificity of each product, and the efficiency of the PCR was determined by analysis of two-fold serial dilutions of cDNA. The PCR efficiency was close to 100%, allowing the use of the 2^−ΔΔCT^ method for the calculation of relative gene expression [13]. All the qPCRs were carried out with negative controls and **Gapdh gene was** used for normalization. The primer sequences were listed in S1 Table.

### DNA methylation analysis

Genomic DNA was isolated from follicles using DNeasy Kits (Qiagen) according to the manufacturer's instructions. Genomic DNA (1 μg) was converted with sodium bisulfite using the EZ DNA methylation kit (Zymo Research, Orange, CA). The PCR reactions were carried out in a total volume of 5 μl using 1 μM of each primer, 200 μM dNTP, 0.2 U HotStarTaq DNA polymerase (Qiagen), 15 mM MgCl_2_, and 10× PCR buffer. One of the two primers in the PCR amplification of the target regions is tagged with a T7 promoter sequence: cagtaatacgactcactatagggagaaggct. This includes ggg transcription start and an 8-bp insert (agaaggct) on the 5′ end (S1 Table). The reaction mix was preactivated for 4 min at 95°C. The reactions were amplified in 45 cycles of 95°C for 20 s, 56°C for 30 s, and 72°C for 60 s followed by 72°C for 3 min. PCR products (5 μl) were aliquoted into 384-well microtiter plates and were treated with 2 μl Shrimp Alkaline Phosphatase (SAP) mix for 20 min at 37^○^C to dephosphorylate unincorporated dNTPs. Subsequently, 2 μl of the PCR reaction were incubated for 3 h at 37°C with 5 μl of Transcleave mix (3.15 μl RNAse-free water, 0.89 μl 5×T7 Polymerase Buffer, 0.24 μl T Cleavage Mix, 0.22 μl 100 mM DTT, 0.44 μl T7 RNA&DNA Polymerase, 0.06 μl RNAse A (Sequenom) for concurrent *in vitro* transcription and base-specific cleavage. The resultant 10 to 20 nl cleavage reaction samples were spotted onto silicon matrix-preloaded chips (SpectroCHIP; SEQUENOM) using a MassARRAYnanodispenser (SEQUENOM) and analyzed using the MassARRAY Compact System matrix-assisted laser desorption/ionization-time-of-flight mass spectrometer (MALDI-TOF) (SEQUENOM). The spectra's methylation ratios were calculated using EpiTYPER software v1.0 (SEQUENOM).

### Plasmid constructions and luciferase assay

The 5'-regulatory region of Hsd3b was cloned from chicken genomic DNA by long PCR (S1 Table). The amplified fragment spans the region between −3544 to +7 bp of the chicken Hsd3b gene, where +1 is the transcription initiation site. PCR products were cloned into the pGL-3 basic luciferase report vector (Promega) using the NheI and XhoI restriction sites. This construct was named −3544 HSD3Bpr-luc. Deletion constructs −2220 HSD3Bpr-luc, −1457 HSD3Bpr-luc, −665 HSD3Bpr-luc, and −215 HSD3Bpr-luc were generated by PCR, with −3544 HSD3Bpr-luc as the template, and confirmed by bidirectional sequencing. Granulosa cells were plated on 24-well plates for transient transfection experiments. The cells were transfected with the luciferase reporter plasmids (800 ng/well) using Lipofectamine 2000 (Invitrogen). Transfection efficiency was normalized by cotransfection of 30 ng of the Renilla luciferase reporter plasmid (pRL-CMV vector; Promega). At 24 h after transfection, recombinant TGF-β1 was added. At 48h after transfection, the cells were lysed and assayed for promoter activity using the dual-luciferase reporter assay system. The enzymatic activity of luciferase was measured with a luminometer (Modulus TM, Turner Biosystems).

### Statistics

Triplicate independent analyses from sodium bisulfite-treated DNA sample were undertaken. Wilcoxon matched pairs test were used for the comparisons of methylation changes in steroidogenic gene promoters between SWF vs F1 follicles and F1vs POF1 follicles. All the real-time PCR and luciferase-reporter experiments were repeated at least four times. Student t-tests was employed to analyze the gene expression levels between follicles as well as the luciferase activity of different truncated Hsd3b promoter-reporters after TGF-β1 treatment. One-way ANOVA followed by Tukey multiple range test was used to analyze Hsd3b gene expression in granulosa cells after different TGF-β1 treatments.

## Results

### Sequencing results summary

High-throughput RNA-seq generated 5.83, 5.66 and 5.89 million raw tags for SWF, F1 and POF1 follicles, respectively. After removing the adaptors and filtering, more than 94% reads were qualified as clean reads. When mapping the clean reads to chicken genome, 80.4%-81.8% reads were successfully aligned, 53.9%-55.2% reads were mapped to gene exons and 49.3%-50.3% reads had unique exon alignments (Table 1). The sequencing reads were submitted to NCBI’s Sequence Read Archive with accession number SRP066743 and the related information are listed in S2 Table.

**Table 1:** Summary of RNA-seq metrics from chicken follicle transcriptomes.

Follicles	Raw reads	Clean reads (clean/all)	Mapped reads (mapped/clean)	Reads mapped to exons (mapped/clean)	Unique reads mapped to exons (mapped/clean)
SWF	5,832,117	5,583,865 (95.74%)	4,489,373 (80.40%)	3,057,117(54.75%)	2,810,752(50.34%)
F1	5,656,555	5,314,713 (93.96%)	4,348,500 (81.82%)	2,865,142(53.91%)	2,618,254(49.26%)
POF1	5,886,919	5,575,816 (94.72%)	4,528,688 (81.22%)	3,078,077(55.20%)	2,778,808(49.84%)

A total of 11,110 genes were detected in three types of follicles together. In the present study, we use "at least a 2 fold difference and FDR≤0.001" as the threshold to screen the differential expressed genes between samples (FDR≤0.001 and |log2Ratio|≥1). When follicles developed from SWF to F1, a total of 784 genes were up-regulated and 1526 genes were down-regulated. From F1 to POF1 follicles, we found 921 up-regulated genes and 356 down-regulated genes (S3 Table).

### Functional analysis of differentially expressed genes

We used GO assignments to classify the functions of DEGs in pairwise comparisons between different developmental stages. In the three GO categories (biological process, cellular component, and molecular function), no GO terms were significantly enriched in the comparison of SWF vs F1. From F1 to POF1 follicles, “non-membrane-bounded organelle”, “intracellular non-membrane-bounded organelle”, “adenyl nucleotide binding”, “adenylribonucleotide binding” and “nucleotide binding” were significantly enriched (p<0.05). The detailed GO analysis of the DEGs was shown in S4 Table. To further explore the biological pathways that were involved in the differentially expressed genes, we performed KEGG analysis of DEGs. The top ten enriched pathways during the transition of SWF to F1 and F1 to POF1 were shown in Table 2. Notably, the “adherence junction”, “ribosome”, “p53 apoptosis” pathways could play important roles in both follicle development and degradation (Table 2).

**Table 2:** Significantly enriched pathways.

Pathway		DEGs with pathway annotation (%)	All genes with pathway annotation (%)	*p*-value
**SWF vs F1**				
1	Adherens junction	39 (2.13%)	154 (1.1%)	3.07E-05
2	p53 apoptosis pathway	32 (1.75%)	127 (0.91%)	0.000169071
3	Ribosome	25 (1.37%)	100 (0.72%)	0.000943309
4	Cell cycle	34 (1.86%)	165 (1.18%)	0.004663671
5	Bladder cancer	15 (0.82%)	59 (0.42%)	0.007889492
6	Focal adhesion	81 (4.43%)	477 (3.42%)	0.00829667
7	Metabolic pathways	249 (13.61%)	1660 (11.91%)	0.009756742
8	Fructose and mannose metabolism	14 (0.77%)	55 (0.39%)	0.009969659
9	Regulation of actin cytoskeleton	87 (4.75%)	528 (3.79%)	0.01380869
10	Arrhythmogenic right ventricular cardiomyopathy	27 (1.48%)	134 (0.96%)	0.01448327
**F1 vs POF1**				
1	Ribosome	19 (1.88%)	100 (0.72%)	9.08E-05
2	TGF-beta signaling pathway	20 (1.98%)	129 (0.93%)	0.000985292
3	p53 apoptosis pathway	18 (1.78%)	127 (0.91%)	0.004676113
4	Protein processing in endoplasmic reticulum	29 (2.87%)	251 (1.8%)	0.008688021
5	Bladder cancer	10 (0.99%)	59 (0.42%)	0.009389407
6	RNA degradation	15 (1.48%)	107 (0.77%)	0.01028756
7	Steroid biosynthesis	5 (0.49%)	28 (0.2%)	0.01057962
8	Thyroid cancer	7 (0.69%)	37 (0.27%)	0.01569848
9	Adherens junction	19 (1.88%)	154 (1.1%)	0.01598464
10	Alanine, aspartate and glutamate metabolism	7 (0.69%)	38 (0.27%)	0.01807276

### Quantitative Real-time PCR analysis

To better validate the sequencing data, various genes related to adherens junction (β-catenin, Cadherin 11, ZO-1), P53 apoptosis (Caspase 8, Trail, Bid), steroid hormone biosynthesis (Star, Cyp11a1, Hsd3b) were chosen and quantified by the real-time PCR method. Results from the real-time PCR experiment supported those obtained from transcriptome analysis and demonstrated similar tendency in up- or down-regulation during the follicular transformations from pre-hierarchical to hierarchical and from pre-ovulatory to post-ovulatory (Table 3).

**Table 3:** Relative mRNA expression of 9 selected genes for comparisons of the SWF vs F1 and F1 vs POF1 follicles in respect to RNA-Seq and real-time PCR.

	SWF vs F1 (F1/SWF)		F1 vs POF1 (POF1/F1)	
Gene	qRT-PCR ^a^	RNA-seq ^b^	qRT-PCR	RNA-seq
**Adherens junction**				
β-catenin (NM_205081)	-1.59 ± 0.23	-2.13 (7.52E-81)	1.84 ± 0.15	1.61 (1.80E-11)
Cadherin 11 (NM_001004371)	3.36 ± 0.20	2.62 (7.61E-12)	-1.87 ± 0.29	-2.19 (7.37E-40)
ZO-1 (XM_413773)	-2.07 ± 0.19	-2.77 (7.58E-11)	6.36 ± 0.48	3.76 (1.26E-12)
**P53 apoptosis signaling**				
Caspase 8 (NM_204592)	-6.77 ± 0.31	-15.67 (6.22E-14)	10.2 ± 0.88	17.27 (0.000261)
Trail (NM_204379)	-3.61 ± 0.37	-2.79 (0.0146)	17.73 ± 1.85	5.28 (2.40E-05)
Bid (NM_204552)	-8.69 ± 0.75	-14.42 (1.75E-269)	1.27± 0.15	1.75 (0.000132)
**Steroid hormone synthesis**				
Star (NM_204686)	2.01 ± 0.15	1.21 (0.337)	-3.36 ± 0.18	-19.56 (5.99E-18)
Cyp11a1 (NM_001001756)	4.41 ± 0.2	1.75 (2.08E-11)	-14.72 ± 0.97	-19.29 (0)
Hsd3b (NM_205118)	2.83 ± 0.21	5.39 (3.17E-12)	-3.41 ± 0.28	-4.99 (4.87E-168)

^a^ Data were expressed as mean of fold change ± SEM.

^b^ Data were expressed as mean of fold change (FDR).

### Methylation levels in promoters of genes related to progesterone synthesis

**Fig 1:**
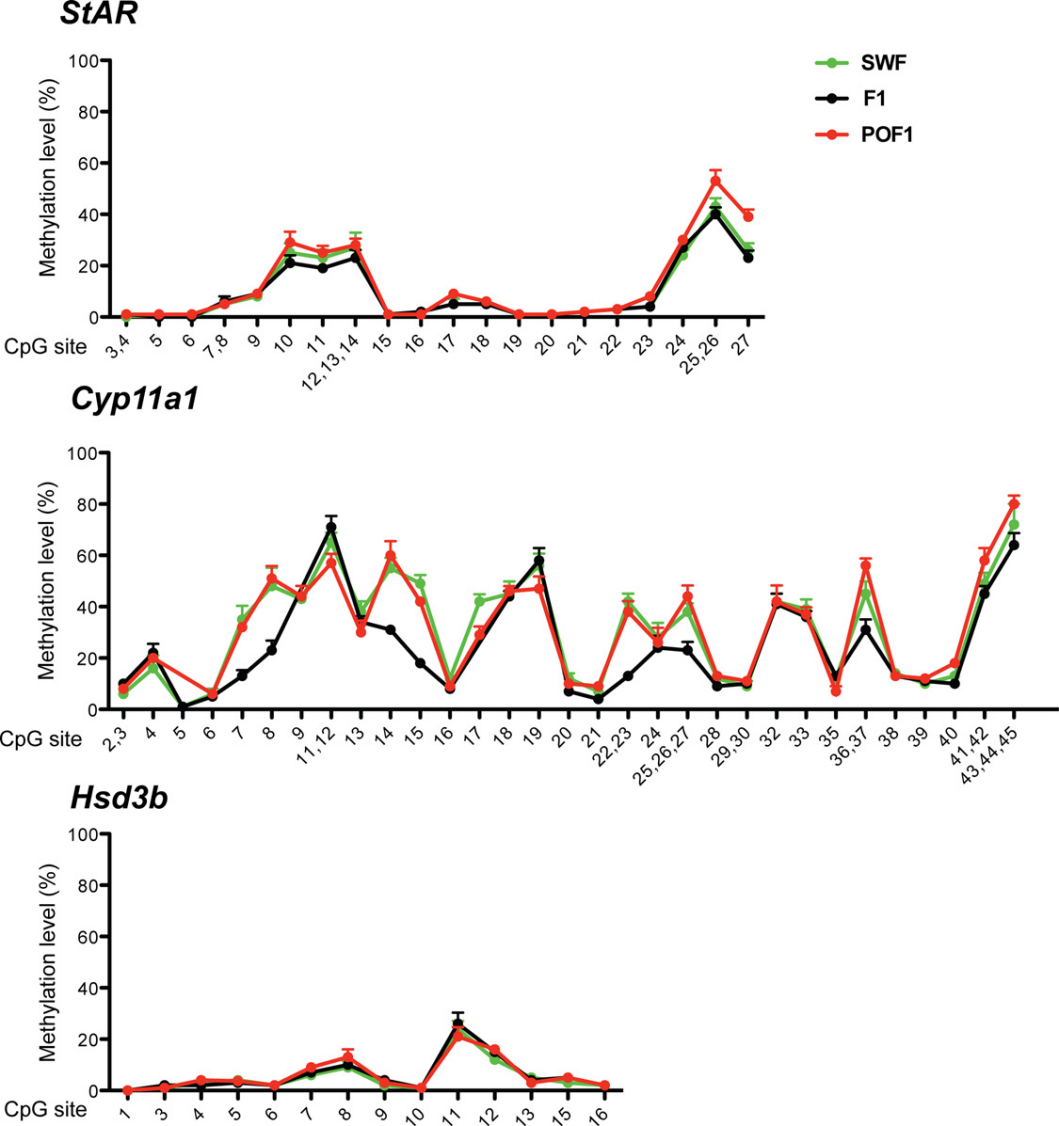
Methylation patterns of the Star, Cyp11a1 and Hsd3b promoters in chicken follicles. Site-specific methylation levels of the proximal promoters of Star, Cyp11a1 and Hsd3b from SWF, F1 and POF1 follicles were compared. The Sequenom MassARRAY platform was used for the quantitative methylation analysis. The CpG units locations are as defined in S1 Fig. Data are expressed as mean ± SEM. n = 3 animals.

Ovarian follicles are able of producing steroid hormones, such as estrogen and progesterone, and this essential ability changes according to their developmental status[14]. The progesterone synthesis is initiated with the transportation of cholesterol into the inner mitochondrial membrane by steroidogenic acute regulatory protein (StAR), then cholesterol is converted to pregnenolone by cytochrome P450 side-chain cleavage enzyme (Cyp11a1), transported out of the mitochondria and catalyzed to progesterone by 3β-hydroxysteroid dehydrogenase (Hsd3b)[15]. The lipid-soluble steroids easily diffuse through cells and the steroids synthesis directly reflects the levels of steroidogenic enzymes, which in turn were regulated primarily through gene transcription[16,17].Our RNA-seq and qPCR results show that Star, Cyp11a1 and Hsd3b were differentially expressed in different chicken follicles and could have undergone an increase or decrease in DNA methylation. For the analysis of methylation status of the Star, Cyp11a1 and Hsd3b genes, several amplicons spanning the corresponding proximal promoter regions were designed. A total of 89 CpG sites were covered and genotyped by using Sequenom Technology (S1 Fig). The methylation status of individual CpGs in proximal promoters of Star, Cyp11a1 and Hsd3b were shown in Fig 1. We found no significant changes in the average methylation frequency of Star promoter between SWF and F1 follicles (p = 0.1878, Wilcoxon matched pairs test), whereas the methylation level of Cyp11a1 promoter was much lower in F1 follicles than in SWF follicles (p<0.0033). Both Star (p = 0.0061) and Cyp11a1 (p<0.0093) gene promoters exhibited significant higher methylation levels in post-ovulatory than pre-ovulatory follicles. However, methylation analysis of Hsd3b revealed that most of the proximal promoter region was hypomethylated in all ovarian follicular samples investigated (Fig 1).The overall DNA methylation patterns of proximal promoters of Cyp11a1 and Star but not Hsd3b were in negative correlation with the respective transcript abundance levels in different follicles (Table 3).

### TGF-β1 inhibits Hsd3b gene transcription in granulosa cells

**Fig 2:**
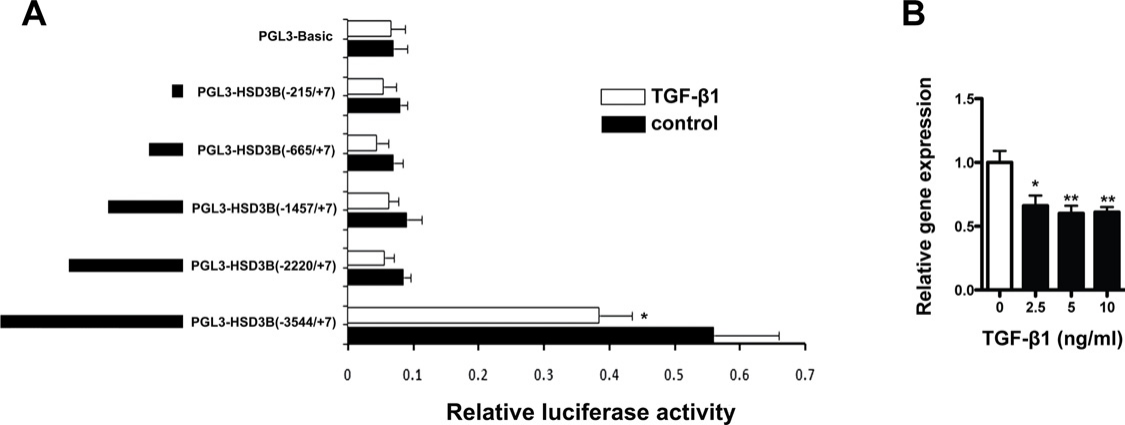
Effect of TGF-β1 on the transcription of Hsd3b gene. **(**A) Granulosa cells were transfected with Hsd3b promoter reporter constructs containing 5′ serial deletions. Twenty-four hours later, cells were treated with TGF-β1 (5 ng/ml). A renilla luciferase reporter plasmid was used as the internal control to correct for transfection efficiency. (B) qPCR shows Hsd3b gene expression was inhibited by TGF-β1. All the data are presented as the mean ± SEM from at least four independent experiments. The housekeeping gene GAPDH was used for normalization. Student t-test was used to analyze the luciferase activity in TGF-β1 treated cells compared to control. One-way ANOVA followed by Tukey multiple range test was used to analyze Hsd3b gene expression in granulosa cells after different TGF-β1 treatments. * *P* < 0.05, ** *P* < 0.01.

The majority of CpG sites in Hsd3b proximal promoter region were unmethylated during the follicle development and ovulation, indicating that this region is important for transcription. Then we analyzed the transcriptional regulation of Hsd3b promoter employing the luciferase reporter assay in primary granulosa cells, in which Hsd3b was exclusively expressed in the chicken pre-ovulatory follicles[18]. The luciferase reporter assay showed no transcription activity in the proximal region of Hsd3b gene in granulosa cells. However, much higher transcription activity was observed in the distal promoter region (Fig 2A). We propose that the core promoter region of Hsd3b gene was located in the distal promoter region and we try to identify the signaling mechanisms that could regulate the promoter activity and gene expression of Hsd3b in different follicles. Among the top enriched pathways during follicle development and ovulation identified by the KEGG pathway analysis, the TGF-β signaling changed significantly between F1 and POF1 follicles and could therefore induce changes in follicular gene expression (Table 2). Among the TGF-β family ligands, TGF-β1 is the main isoform expressed in chicken pre-ovulatory follicles[19].Therefore, we transfected the granuloma cells with different promoter-reporter plasmids and then treated the cells with TGF-β1. We found that TGF-β1could significantly inhibit distal but not proximal promoter activity of Hsd3b gene (Fig 2A), indicating that the promoter region from –3544 to –2220 bp of the chicken Hsd3b gene should contain the TGF-β1 responsive element. Accordingly, the gene expression level of Hsd3b in cultured granulosa cells was inhibited by different doses of TGF-β1 treatment (Fig 2B).

## Discussion

Efficient ovarian follicular development and ovulation is necessary for egg production in poultry industry. Gaining insight into the gene expression patterns during ovarian follicle development will benefit the improvement of the laying performance as well as the better understanding of avian reproductive physiology. In the present study, each follicle stage displayed a very unique transcriptome and the identified DEGs whose transcriptional regulation was correlated with the pre-hierarchical to hierarchical and pre-ovulatory to post-ovulatory transitions are candidate regulators of these key developmental processes.

The follicle growth and regression require continuous cell interactions and re-arrangements which involve the intercellular junction and cytoskeletal architecture alterations. The present RNA-seq data suggested the adherens junction characteristics changed the most from SWF to F1 follicles, which indicated that cell-cell adhesion in pre-hierarchy follicles could provide the growth triggers for the selected follicles[20]. The cadherin-catenin complex constitutes the core structure of the adherens junctions at the cell surface[21].Our results showed that the classical N-cadherin but not E-cadherin was present in the cells of chicken follicular wall prior to ovulation, which is in agreement to the observations in rodents[22,23]. Another two unconventional cadherins, cadherin-11 and -13, were up-regulated from pre-hierarchy to hierarchy follicles. After ovulation, all the expressed cadherins reduced rapidly and hence resulted in the loss of adherens junctions in the loosened granulosa and theca cells in the transformed POF follicles[24]. Quite unexpectedly, the expression level of β-catenin, the classical cadherin adhesion partner, was inversely correlated to the main cadherins. β-catenin is also the key transcriptional co-activator of the Wnt signaling pathway[25], therefore the reduction of β-catenin in F1 follicles is consistent with the evidence that Wnt/β-catenin could inhibit the follicle maturation[26] and ovulation [27,28]. Given the importance of intercellular junctions and cytoskeleton in cell shape change during follicle selection and growth, we hypothesized that a better understanding of cell-cell adhesion and cytoskeletal architecture in different follicles would help identify candidate growth triggers and regulators of follicle development.

The chicken ovarian follicles are unique dynamic cellular structures that efficient and consistent cell death and survival occur. The present data indicate that the apoptosis pathway was actively involved in follicle development and regression. Follicular cells from chicken pre-hierarchal follicles are susceptible to apoptosis and the follicles are easily undergoing atresia[29]. By comparison, follicles selected to enter the pre-ovulatory hierarchy rarely undergo atresia and the granulosa cells are highly resistant to apoptosis[30,31]. Similar to mammalian corpus luteum, the post-ovulation follicles degenerated mainly through apoptosis process [32,33]. The comparisons of follicle cells from different phases of the ovulation cycle could enable us to identify the cell death or survival factors in the follicles. The progression of apoptosis can be divided into phases of initiation, execution and termination[34]. In agreement with previous studies, the present data showed that both intrinsic (Bid) and extrinsic (Trail) pro-death initiation signaling were repressed in pre-ovulatory follicles[30,35]. During follicle development, the initiator caspase (caspase8) was the mostly down-regulated member of the caspase family, whereas the expression of executioner caspase (caspase 3, 6) remained stable which confirmed previous observations[35,36]. Therefore, we speculate that the pre-hierarchical follicle atresia was largely controlled by the initiation phase of apoptosis since the execution machinery was equally functional in pre-hierarchical and hierarchical follicles. Furthermore, the expression of genes encoding the key apoptosis initiators and executors as well as several caspase inhibitors reached maximum in POF1 follicles, demonstrating the progressive apoptotic activity in the post-ovulatory follicles should accelerate their elimination[32]. Follicular development, atresia and regression seem to depend upon a sophisticated balance between the death and survival factors. Elucidation of the molecular mechanisms and hormonal regulation of the suicide program in ovarian follicular cells should help in the design of new therapeutic strategies for the treatment of ovarian disorders characterized by excessive cell degeneration or growth, such as granulosa cell tumor[37].

The steroid progesterone is produced mainly by corpus luteum or placenta in mammals, whereas in avian species the pre-ovulatory follicles are the major tissue responsible for progesterone synthesis[38]. Progesterone participates in the regulation of important reproductive processes in chicks such as ovulation, oviposition, sexual and nesting behaviors[39]. Researchers previously found that the steroidogenic activity was minimal in non-hierarchical SWF follicles[40,41], and then increased and reached the maximum in pre-ovulatory F1 follicles[42,43]. Accordingly, we observed elevated expression levels of the genes (Star, Cyp11a1, Hsd3b) encoding the key enzymes for progesterone synthesis in F1 follicles (Table 3), which is in consistent with previous studies[44–46]. In mammals, the capacity of progesterone synthesis reaches the maximum in corpus luteum, in which the highest expression levels of Star, Cyp11a1 and Hsd3b were found[47]. In chicken, the gene expression of Star, Cyp11a1 and Hsd3b reduced significantly immediately after ovulation (Table 3)[48], although the post-ovulatory follicles maintained certain steroidogenic activity for a short period [24,49,50]. Therefore, the peri-ovulation stage features a unique steroidogenic gene expression profile that is clearly different from both the preceding and subsequent stages, which prompted us to further investigate the transcriptional regulation of the key genes for progesterone synthesis in chicken follicles.

Much progress has been made in resolving the complex hormonal signals and transcription factors that regulate the Star, Cyp11a1 and Hsd3b expression in different tissues among different species[15]. In addition, recent investigations also showed that epigenetic modifications, such as DNA methylation, could also contribute to the transcriptional regulation of Star and Cyp11a1 in different cells[51,52]. We also found the evidence that DNA methylation was involved in differential expression of Star and Cyp11a1 but not Hsd3b in chicken follicles. Particularly, the methylation pattern of Cyp11a1 promoter was reversely correlated with the gene expression in different follicles, indicated that DNA methylation in Cyp11a1 could be an essential mechanism by which the follicular steroidogenesis is regulated. However, the DNA methylation levels of bovine Cyp11a1 and rat Star proximal promoters does not change during ovulation and luteinization despite a profound gene expression change was noted simultaneously[53,54]. Nevertheless, it appears that DNA methylation regulation of Hsd3b was relatively conserved among species since its proximal promoter region remained unmethylated (<20%) during ovulation in both cattle and chicken[54]. Our data, however, do not exclude the possibility that a distal region of the Hsd3b promoter not evaluated might be methylated to silence the gene in post-ovulatory follicles. It would be interesting to examine whether other epigenetic mechanisms, such as changes in histone modification and chromatin structure, could be also involved in the transcription regulation of Hsd3b in different follicles similar to mammals [55].

The steady low level of DNA methylation in proximal region of Hsd3b promoter in different follicles favors gene expression by making the *cis*-elements accessible or responsive to external signals. However, the present promoter-reporter analysis showed that the proximal promoter region has limited basal transcriptional activity in follicular granulosa cells cultured *in vitro*. Therefore, it appears that the circulating hormones or follicular cytokines are necessary to induce and maintain Hsd3b expression in pre-ovulatory follicles *in vivo*. We found TGF-β1 could inhibit Hsd3b expression by suppressing the promoter activity in chicken follicular cells, which is in agreement with both the granulosa and theca cells from bovine follicles[56,57]. It seems paradoxical since the endogenous Hsd3b gene expression reached the highest in the F1 follicles, in which the different TGF-β ligands are enriched [58]. The TGF-β family ligands have been found to play diverse roles in the ovary [58,59] and the signaling of TGF-β is finely regulated at multiple levels [60]. Whether individual TGF-β family ligands act differently in Hsd3b regulation or this inhibition is outweighed by other predominant signaling mechanisms in avian follicular steroidogenesis requires further study.

## Conclusions

Our current transcriptome database will be of great value in revealing molecular and cellular signaling associated with follicle selection and ovulatory events in the chicken ovary. The DNA methylation analysis in genes related to progesterone synthesis highlighted the conservation and evolution of molecular mechanisms underlying epigenetic regulation of follicular steroidogenesis among different species.

A complex combination of epigenetic modifications and transcription factors along hormone-activated signaling pathways obviously direct the activation and de-activation of target genes in the chicken follicles.

## Supporting Information

S1 Fig Map of the Star, Cyp11a1 and Hsd3b proximal promoters and CpG sites. Individual CpG sites were underlined with solid line and numbered. Arrows indicate the primer binding sites for the PCR amplicons. The putative transcription start site was denoted as +1. The star codon is in red.
(PDF)

S1 Table Primers used in this study. (DOCX)

S2 Table Raw data of RNA-seq analysis. (XLSX)

S3 Table The differentially expressed genes in SWF vs F1 and F1 vs POF1 follicles. (XLSX)

S4 Table Gene Ontological classification of differentially expressed genes. (XLSX)
