# Supplementary material for: Dynamic Changes in the Follicular Transcriptome and Promoter DNA Methylation Pattern of Steroidogenic Genes in Chicken Follicles throughout the Ovulation Cycle
Source: PLoS One. 2015 Dec 30;10(12):e0146028. doi: 10.1371/journal.pone.0146028 (PMC4696729; doi:10.1371/journal.pone.0146028)
Supplement: S1 Fig — Individual CpG sites were underlined with solid line and numbered. Arrows indicate the primer binding sites for the PCR amplicons. The putative transcription start site was denoted as +1. The star codon is in red. (PDF) [file pone.0146028.s001.pdf]

Star

GCTGAATGTC AAAAGGGAAG TGAGGTCCCT GGGTGAGTAA GGGACGTGCA GTGGGGCTGA

GGGGGCTGGG ATGGGGCCAG GACCCCCCTT TCTTGCAGCC CCTGGGCGCG GTGGTGGTGT

GGGACACCTG AGGAAGTGCT GATGTGGCAC TGTGGAGCCG GCAAACATCC AGGGACACCT
F1 1

CGGTTCTTCA GGTATCCTGA AGTTTTTGCT GTGTCCCTGG AACAGTGGCA CCCCGGAGCC
2 3 4

GAAGGGGTGG CTCTGTCAGA GATCAGTTCA GTTCTTCTCA ACAACAAGTG CATGTCCTTG

CTCCGAGGCC GTGTGTTCAG GGCGGCCGCT CCATTGGGAT AACAGCAGTG CAGAGCTGGA
5 6 7 8

GATCTCTGTG CTGGGGAGAG CTGTGCCTGT GCTGCTGCAT TACCAGGGCA GCGATTTGGG
9

AAGGAGATGC TCAGTTGCTG CCTTCCCCCG GTGCCTCCAA ACCCCGTCCC GTTGCATGTG
10 11

AGATGTGCTG TGCTGCGCGC AGTCGGGGTG GAGAGGATCC ATCCAGATCC ATCCATCCAT
12 13 14 F2

CCTGGAGCTG TGGGCAGGAG CAGTGCGGCC CTTCCTGGGC ACAGGTAGGG CTGAGCACCA
R1 15

CGAGGCTGTG CTGCAGTTCC CCGTGTGCCC TCAGCACCAC AGCGAGTGGG TGACCTCCCC
16 17 18

CCTCACCAAG TTGTTGCGAC AGCAAAGAGG AGCAAGGTCA GCATCCCTGC AGCAGAGTGC
19

AGGAGCTGAC GTCAAAGGAA GCAGCTGTGA TTCACAGCTT TTGGGGAGCG CTCAGTGCTG
20 21

GGGCTGTAAC TCATTCCACA GATGTCATTT CAGCCTTGAG CGTTATCACT GCTATTGCTC
22

CACCCCTGGG GAGATGTGGG GCTGCCCCCT CAGTGCAGCA GAGTGTGGCT GGGTGGGACC

CCCACCCTGC AGCCTCCCTA TGCACCCTGC AGCCCTGCTC TGCATGCACA GCCCTCAGCT
F3 R2

GCAGCTCCAC GCAAGCCGTC TACAAAATGC TCTCAGCCTC ACAACGTGCG CAGAGCATCC
23 24 25 26 27

GTCTGCATTC CCCACGTGCT GTGCAGCAGC AGAGGGGGGC AGCAGAGTTT GCCCCACGTA
28 R3

TCTGGGCAGC AGCAGCTGCA TCAGGATGTG CGCATGCGCT GCTGCTGGCA CCCACAGCGA
+1

CCCACAGCCC CCGGCAGATG CATGGGGAGG TGGGGCTGTC CTTGGCCCCC

Cyp11a1

```
AGGTTTGGTG TAGGGCATTG ATTTGACCTC CCCTGTGATA AATCACTGCA CTCCCAGCAC

CGGCTGCTCC AGATCCCCGG GAGACGATGG GGGTTTTCCA CACATCTGGG TTCTGTGGGC

TGTGTGGGGG CGAAGGTTTC ACCCCCAGAT TGGGGTTTTC CCAGCATCTC CTGCTACGTA
              F1                                              1
AAGTGTGATT TTGGGGGGGA AGTGTGATGG GGTGGTCTTT AGTCACCCTG CTCATAGGGG

CGCGGAATGA AGAAAGCCCC ACAGGTGGAA ACTGACAGCG TAATGCCCAC GTTGGGCTGG
 2 3                                     4            5   F2
AAGGATCCTA CATATGTGTC ATGCGGAAGA GCCCCTTTGG CATGGGGGTC CCATCTGTCA
                         6
TCATCGATAC CACGGGAAAA CACGGTCACA GCCCTGTCCC CTTAGGGGAA GCCCCGTTTC
    7        8          9                           R1     10
CAACCCCCTC TTTGTCACAA AGACCCCGAA CCGCTGCATT TCTCGTACCC AAGTGCCGTA
                            11    12           13            14
AGCAACAGGA AGCGATGGTC CAAACGCCCC CTAAGGGCCG TGTTTTGGAG GGTCCGCTGG
             15           16             17                18
GACCCGACAA GTGTCGGGGA GGGGACAGGT GGCCGTGTTG GTGGCAGTGG ATGGGACAGG
    19         20                   21
CGCGCCCCCC CGTGCTCAGC GCTGCGCTCA GCGGTTTTGC TCCTGGAGCG GAAGGAATAG
22 23      24        25   26      27                28
GGGCTGAATG AAACCGAAAC GCTAAACGAA ATCTGCTGAG TCCCCACTTC GGGAGCTCAG
               29    30     31
CAAATCCCCG TTGGGCGGCT GCTCCCATCC ATCGCCCCAG GAACCTCTCA GGGCCACCTC
        32      33                 34    F3
TGCAGGCTGT GCCTTCCGAA GCTGAGCTGG GAGGGGGGGG GAAGGGGTGG GTGGAACAAG
       R2        35
CAGCACCGCG AGCACTTTTA TGATTCATTT TGCAGGGAAA GCAAAACATT TTGAAGGCGG
      36 37                                                   38
GGAAGCTCGT ATTTATGTTG GAATGGCTTT GATTGAGGTT GCCCGGGCTG CCAGCCAATG
       39                                      40
GCCCAGCAAC AGCGCGGGGC AGGAGCACGC TCCCCGAGCG CTGTCCCCCA AAGCAAGGCC
             41 42           43      44  45                  R3
ACCCCATCCC CCCCCCATCC CGACCCTGTT TGGGGCATTT CGCCAGCACT CGGCCCCGTG

ACGTGCTGGA GGCGGGCGAG AGCTCCTCGC CTTGAGCCCA GGATTCAGGG ATAAGAGCGA
                      +1
GGTCGCCCCG GCCGTGCGCT CGCCCCGCTC CCATGCTCTC CAGGGCTGCA CCCATAGCGG
```

Hsd3b

TGCCAAAATT TCATCATCTG TGTCAGCCAG AAGCTCTCTT CTCCTGGACC AAAGATGGGT

TTGTTTTCCT GTTGTTATTT GGAGGCACAG TTTTCCAGCA GTGAAGTTTC TAAAGCTTTT

F1

CTTCAAGGAC CTCTTTCGTG AGTGAACAAA AATCACACCC ACAGAATAAA ACAGGGAGTT

1

TAGGAGTGTT TAGCTGCAAA AAGACAATGA AGCATTTTAA AAGATCCACT CCAGCTTTTC

AGCAATTCCA GCATAATTAG GTGTGCTGCT GGGCTTCTCT GTAATATCCC TCGGCAGATT

2

TTAAATGGCT ACCTGTTCAA CATTTTGCTT CAGTCTTACT ATCCGTGATT GAGAAAGAGG

3

AAAGGTCCGG GCATCCTGCC AGGACACAAC AACCTGCGAA GGCAATGAGG ACAGTGTGCA

4 5

TTTCAAGCTG GCGCCTAAGC CCTATCCCAG TCCCACCCTT GGAATGAATT GTGCTAAAAC

6

TGAGATGAAA CCCTAGTGAG AAGTAACTGA GCGTTTCCCC CTTTTTCTGG TGATGTAGAA

7

AATAAAGACA CCATTCACTG TGAAGCCAGC CACACCCTTT ACTTTAACAG ACATTCCACA

F2

CCAGACATTT CAGAGCTGAG CCCTTCCAGC AGCTTTATTT TAACCACTCT ATACTTCAGA

R1

TCATTTCATC GCTCCAAAAG CCTCGCATAT GCCAAACAAA TACAAAATCT CCTGTTTCGC

8 9 10

ACAGAGTGAG AAATCCCTAC CATAATGGGG AGCTCAGCAT CCAAAAAGAG TCCACAGAGT

AAACAGCTAT TTCTTCTATT TACATTTAGA TTTAAAGTTC TCTCCCTTAT TACTGTTACA

GAAGCAAGCA CAGAAAGCGA ATGATCAGAA CAAGGATTTT TGCTCCTAGG ATATAGGACC

11

AGGTCTAATC GGGTGTTCAG ACACCATTGC ATCAAGGACG TTCCACTCCG AAAGAGGAGG

12 13 14

GGTGGAAGTA CCATATTTGT TTGTAGCAAA GGAGACGTGT GAGAGGGAGG GCAATCAAAG

15

TGGTGTGGTA CATAAATACA GGCGAATCTC CTCCACAGCC TTCTGAATGC TAGAGAGGAG

16 R2

GAACTGAAGC GGACATCGGT GGGGTTTGTT TAGCACTGAG GTCGAGCACT TTCAGATATG

+1 1st intron

TTAACATCCT CAGTTTGTAA TAGTTCATAA ATAGCTTTTA CTTTGCATGG GAAAGTAGCA
