# Supplementary material for: Dynamic Changes in the Follicular Transcriptome and Promoter DNA Methylation Pattern of Steroidogenic Genes in Chicken Follicles throughout the Ovulation Cycle
Source: PLoS One. 2015 Dec 30;10(12):e0146028. doi: 10.1371/journal.pone.0146028 (PMC4696729; doi:10.1371/journal.pone.0146028)
Supplement: S1 Table — (DOCX) [file pone.0146028.s002.docx]

| Experiment | Genes | Sequence |
| --- | --- | --- |
| Real-time PCR | β-catenin (f) | TTGTGCGTACCATGCAAAAT |
|  | β-catenin (r) | ACACAGAATCCACCGGAGAC |
|  | Cadherin 11 (f) | ACAGCCTGAAGGTAGAGGCA |
|  | Cadherin 11 (r) | TTGCAGCATCAGGATCTTTG |
|  | ZO-1 (f) | GAGCGCAAGTTTGAAAGTCC |
|  | ZO-1 (r) | AGGAGGCTGTGATGAGCTGT |
|  | Caspase 8 (f) | GGTGAGCAGCAAGATTGACA |
|  | Caspase 8 (r) | CTGCCTCTGCTCCCATTTAG |
|  | Trail (f) | AGCCTGACTAAACCTGGGGT |
|  | Trail (r) | GCAGCTGTTTTTATGTGCGA |
|  | Bid (f) | TACAGCGTGTCTTCAGCACC |
|  | Bid (r) | CTCTGCCTTCCCTTTCACAG |
|  | Star (f) | GAACCCCAGCGTCAAAG |
|  | Star (r) | GCACCGAACACTCACAAA |
|  | Cyp11a1 (f) | CAGGGGGACAGGGTGAA |
|  | Cyp11a1 (r) | AGCGTCTTGGGGGGGAT |
|  | Hsd3b (f) | CTTCCATCATTGACACC |
|  | Hsd3b (r) | TCAGCACACATTCCTCT |
|  | Gapdh (f) | GAGAAACCAGCCAAGTATGATG |
|  | Gapdh (r) | CACAGGAGACAACCTGGTCC |
| DNA methylation massARRAY | Star (F1) | aggaagagagATTTGAGGAAGTGTTGATGTGGTAT |
|  | Star (R1) | cagtaatacgactcactatagggagaaggctTACTCCTACCCACAACTCCAAAATA |
|  | Star (F2) | cagtaatacgactcactatagggagaaggctATAAAAAAAATCCATCCAAATCCAT |
|  | Star (R2) | aggaagagagGGGTTGTGTATGTAGAGTAGGGTTG |
|  | Star (F3) | cagtaatacgactcactatagggagaaggctTACAACCTCCCTATACACCCTACAA |
|  | Star (R3) | aggaagagagGTGGGGTAAATTTTGTTGTTTTTTT |
|  | Cyp11a1 (F1) | cagtaatacgactcactatagggagaaggctAAAATTTCACCCCCAAATTAAAA |
|  | Cyp11a1 (R1) | aggaagagagGTTTTTTTTAAGGGGATAGGGTTGT |
|  | Cyp11a1 (F2) | aggaagagagGGGTTGGAAGGATTTTATATATGTG |
|  | Cyp11a1 (R2) | cagtaatacgactcactatagggagaaggctCACAACCTACAAAAATAACCCTAAAAA |
|  | Cyp11a1 (F3) | cagtaatacgactcactatagggagaaggctAAAAACCTCTCAAAACCACCTCTAC |
|  | Cyp11a1 (R3) | aggaagagagTGGTTTTGTTTTGGGGGATAG |
|  | Hsd3b (F1) | cagtaatacgactcactatagggagaaggctATTTCTAAAACTTTTCTTCAAAAACCTC |
|  | Hsd3b (R1) | aggaagagagAAGTAAAGGGTGTGGTTGGTTTTAT |
|  | Hsd3b (F2) | cagtaatacgactcactatagggagaaggctAAACATTTCAAAACTAAACCCTTCC |
|  | Hsd3b (R2) | aggaagagagTTAGTATTTAGAAGGTTGTGGAGGAGA |
| Luciferase-reporter | Hsd3b (f) | ctagctagcTGCCCTGCCTTACTACCAC |
|  | Hsd3b (r) | ccgctcgagCAAACCCCACCGATGTC |

**S1 Table. Primers used in this study.**
